# Forensic Engineering of Advanced Polymeric Materials—Part VII: Degradation of Biopolymer Welded Joints

**DOI:** 10.3390/polym12051167

**Published:** 2020-05-19

**Authors:** W. Sikorska, M. Zięba, M. Musioł, M. Kowalczuk, H. Janeczek, P. Chaber, O. Masiuchok, V. Demchenko, V. Talanyuk, M. Iurzhenko, J.E. Puskas, G. Adamus

**Affiliations:** 1 Centre of Polymer and Carbon Materials, Polish Academy of Sciences, 34. M. C. Skłodowska St., 41-800 Zabrze, Poland; wsikorska@cmpw-pan.edu.pl (W.S.); mzieba@cmpw-pan.edu.pl (M.Z.); mmusiol@cmpw-pan.edu.pl (M.M.); marek.kowalczuk@cmpw-pan.edu.pl (M.K.); hjaneczek@cmpw-pan.edu.pl (H.J.); pchaber@cmpw-pan.edu.pl (P.C.); 2 International Polish-Ukrainian Research Laboratory ADPOLCOM, Centre of Polymer and Carbon Materials, Polish Academy of Sciences, 34. M. C. Skłodowska St., 41-800 Zabrze, Poland; 3 E.O. Paton Electric Welding Institute of the National Academy of Sciences of Ukraine, 11. Kazymyr Malevych Str., 03680 Kyiv, Ukraine; omasiuchok@gmail.com (O.M.); dvaleriy@ukr.net (V.D.); vika-toritalanuk@ukr.net (V.T.); 4ewip@ukr.net (M.I.); 4 International Polish-Ukrainian Research Laboratory ADPOLCOM, E.O. Paton Electric Welding Institute of the National Academy of Sciences of Ukraine, 11. Kazymyr Malevych Str., 03680 Kyiv, Ukraine; 5 Department of Food, Agricultural and Biological Engineering, The Ohio State University, 1680 Madison Avenue, Wooster, OH 44325, USA; puskas.19@osu.edu

**Keywords:** biopolyesters, polylactide (PLA), poly(3-hydroxyalkanoate) (PHA), polyester welded joints, hydrolytic degradation, differential scanning calorimetry (DSC), electrospray ionization mass spectrometry (ESI-MS)

## Abstract

Welding technology may be considered as a promising processing method for the formation of packaging products from biopolymers. However, the welding processes used can change the properties of the polymer materials, especially in the region of the weld. In this contribution, the impact of the welding process on the structure and properties of biopolymer welds and their ability to undergo hydrolytic degradation will be discussed. Samples for the study were made from polylactide (PLA) and poly(3-hydroxyalkanoate) (PHA) biopolymers which were welded using two methods: ultrasonic and heated tool welding. Differential scanning calorimetry (DSC) analysis showed slight changes in the thermal properties of the samples resulting from the processing and welding method used. The results of hydrolytic degradation indicated that welds of selected biopolymers started to degrade faster than unwelded parts of the samples. The structure of degradation products at the molecular level was confirmed using mass spectrometry. It was found that hydrolysis of the PLA and PHA welds occurs via the random ester bond cleavage and leads to the formation of PLA and PHA oligomers terminated by hydroxyl and carboxyl end groups, similarly to as previously observed for unwelded PLA and PHA-based materials.

## 1. Introduction

Biodegradable polymers are the most developed class of new sustainable materials, leading to numerous technological innovations. In recent years, it has become extremely important to design polymer packaging materials that will be safe for human health and the environment, and to find new areas in which their unique properties can be adopted [1]. The largest market of environmentally responsible and sustainable polymer materials is the packaging industry (39%), with construction and transportation (including ground and air) also being important [2]. PLA (polylactide) and PHAs (polyhydroxyalkanoates) are the most broadly tested and developed biopolymers. PLA is a biodegradable thermoplastic aliphatic polyester, whose monomer is derived from renewable resources, such as corn starch, cassava roots, starch or sugar cane [3]. Poly 3-hydroxyalkanoates belong to a family of fully biodegradable polyesters produced by several strains of bacteria for carbon and energy reserves [4,5,6]. The properties of PHA biopolymers vary from crystalline-brittle to soft-sticky materials depending on the chemical structure of the side aliphatic chain attached to the β-carbon [7]. The processability of PHB biopolyester is comparable to that of conventional thermoplastics polymers [8]. Many practical applications, taking advantage of the biodegradability of the PLA and PHAs, especially poly(3-hydroxybutyrate) PHB and its copolymers with (R)-3-hydroxyvalerate (PHBV), have been suggested, including for use in agriculture and as biodegradable packaging materials [9,10]. Other areas of application include construction, packaging, medical, electronic, etc. [11,12,13,14].

These applications usually require different joining methods of the polymeric materials. Welding could be considered the most effective method for thermoplastic polymeric materials such as PLA and PHB. The process of welding includes activation of the two surfaces, usually by heating them to a temperature higher than the melting point of a polymer, and joining the melted surfaces through the use of force. The most popular welding methods used in practice are heated tool welding, resistance welding, and ultrasonic and high-frequency welding. Butt and overlapping welding are also multipurpose methods that can be used for PLA and PHB biopolymers [15,16]. Klinstein et al. reported the weldability of PLA using two different methods: impulse high frequency and ultrasonic welding. Additionally, they investigated the impact of the weld joints on the mechanical properties of the welded material. It was shown that in the case of impulse welding, excessive weld energy and long heating time with high current values led to film damage and low weld strengths. In the case of ultrasonic welding, the weld strength was proportional to weld distance and inversely proportional to weld/melt velocity [17]. Pagano et al. reported that it is possible to join PLA to aluminum thin films for food-packaging applications using laser transmission welding. The joint quality depends on the power level of the laser source [18].

Although the influence of processing methods (injection molding, extrusion, foaming as well as fiber spinning and 3D printing) on the properties of the final products of PLA and PHA have previously been studied, the properties of welded products made of PLA and PHA polyesters have not been investigated in detail. Due to the fact that PLA and PHA are the most widely known biodegradable polyesters, and are currently produced on a large industrial scale, information on weld properties would be very important from an industrial application point of view.

Herein we report the results of research on welding of PLA and PHA using various methods. In particular, this work is focused on the structural and molecular characterization of welded PLA and PHA joints, as well as on the investigation of changes in the stability of biopolyester welds during hydrolytic degradation process depending on the welding methods used. To characterize the resulting biopolyester welds and to monitor their hydrolytic degradation, ^1^H Nuclear Magnetic Resonance spectroscopy (NMR), Gel Permeation Chromatography (GPC), Differential Scanning Calorimetry (DSC), and Electrospray Ionization Mass Spectrometry (ESI-MS) have been used. Our approach is consistent with the concept of forensic engineering of advanced polymeric materials (FEAPM), which can help to understand the relationships between the structure of the biodegradable polymer material used, its properties, and behavior for practical applications [19].

## 2. Materials and Methods

### 2.1. Starting Materials

PLA—commercial polylactide type 2002D (NatureWorks^®^, St. Louis MO, USA) with melt flow rate (MFR) of 5–7 g 10 min^−1^ (2.16 kg, 210 °C), density (d) 1.24 g cm^−3^ and content of D mers below 3.5%.

PLA filament produced by the Monofilament Fabric Factory (Jiangmen City, China) [20]. PLA filament—commercial 3D printing filament of 1.75 mm diameter, processing temperature ≤205 °C) with a weight-average molar mass *M_w_* = 274,000 g/mol, molar-mass dispersity *M_w_*/*M_n_* = 2.4 and D-lactide content equal to 5.2%.

The ENMAT Y1000 PHA was supplied by Tianan Biologic Material Co., Ltd. (Ningbo City, China), density (d) 1.25 g cm^−3^, melting points 170–176 °C [21] and used as received.

### 2.2. Description of The Products Samples Made of PLA and PHA Which Were Used to Make Welded Joints

Sample PLA 1—the food container made from PLA rigid film. PLA rigid film was prepared from commercial polylactide type 2002D by the IMPiB Institute in Torun, Poland and processed by vacuum thermoforming into a compostable food container. The procedure was described in Ref. [22]. During thermoforming, the temperature of the heating zones was set at 240 °C for all central zones and 280 °C for external zones. The holding time at lower pressure was 8 s and the total duration of thermoforming was 45 s. A temperature of 50 °C was reached after 15 s. The dimensions of the form nest were 30 × 120 × 155 mm. The average thickness of the side wall of the product was 0.25 mm.

Sample PLA 2—3D printed PLA specimen was formed using the 3D-printer (Sygnis Flash Forge Creator Pro). The Fused Filament Fabrication (FFF) 3D printing technology was used to prepare specimens with 24 mm^2^ cross-section. The parameters of the 3D printing process are given in Table 1.

Sample PHA 3—specimen from ENMAT Y1000 PHA. The sample was formed under the following conditions: PHA powder was placed in the press and a preheated to 140 °C and then heated for 1.2 min without pressure. Next 70 MPa pressure was applied for 1.5 min and the sample was heated to 180 °C for 1 min under the same pressure.

### 2.3. Description of The Welded Joints and Method Used for Obtaining Thereof

***1W—sample of ultrasonically welded PLA 1 (food container)***

One part of the PLA 1 sample was placed on top of another part of the PLA 1 sample and then welded using the ultrasonic welding method (22 kHz) at 1320 W for one second at 3 bar pressure.

***2W—sample of butt welded PLA 2 after cutting it in half obtained with the heated tool method***

Welding conditions: heating temperature 220 °C, duration 65 s, 0.15 MPa pressure.

***3W—sample of butt welded of two parts of PHA 3 pressed specimens obtained with the heated tool method***

The weld was obtained by the connection of the two parts of PHA pressed specimens (sample PHA 3) and butt welding using a heated tool at 200 °C for 5–7 min.

### 2.4. Hydrolytic Degradation of Welded PLA and PHA Samples under Laboratory Conditions

The samples were incubated in 25 mL of distilled water in screw-capped vials with air-tight PTFE/silicone septum. The hydrolytic degradation was conducted for 365 days (one year) in a laboratory oven at 70 °C (±0.5 °C), according to ISO Standard 15814:1999 (accelerated degradation test conditions). After specific incubation times (14, 42, 84, 182, 270 and 365 days), samples were taken [23]. 

### 2.5. Measurement Methods

#### 2.5.1. Differential Scanning Calorimetry (DSC) Analysis

DSC analyses of the starting materials, samples before welding (PLA 1, PLA 2 and PHA 3) and their corresponding welds (samples 1W, 2W and 3W, respectively), as well as PHA 3 after 9 months of hydrolytic degradation, were conducted using a TA-DSC Q2000 apparatus (TA Instruments, Newcastle, DE, USA) as described previously in [24]. The calorimetric traces were acquired from −50 °C to 200 °C at a heating rate of 20 °C min^−1^, under a nitrogen atmosphere (flow rate = 50 mL/min) using a sample size of 10 mg. The instrument was calibrated with an indium standard at calorimetric precision of ±0.05%. The glass transition temperature, T_g_, was taken as the midpoint of the heat capacity change. The melting temperature, T_m_, was taken as the peak maximum of the melting endotherm. The degree of crystallinity, X_c_, of the PHA was calculated using the following equation:

X_c_ [%] = ΔH_m_ 100/ΔH_o_

where ΔH_m_ is the experimental melting enthalpy of PHA (in J/g) and ΔH_o_ is the literature value of the melting enthalpy of 100% crystalline PHB (164 J/g) [25].

#### 2.5.2. Determination of pH Changes

The pH of the degradation medium was measured using a Mettler Toledo SevenMulti S40 pH-meter (Mettler Toledo, Columbus, Ohio, USA) at T = 23.0 ± 2.0 °C according to reference [26]. The pH-meter (resolution: ± 0.1 mV, ± 0.001 pH units) was equipped with an InLab Science Pro 3-in-1 electrode featuring an ARGENTHAL™ reference system with an Ag+ trap and reference inner electrolyte composed of 3 molL^−1^ KCl (Metrohm, Switzerland). The electrode was calibrated using buffers (Mettler Toledo, Switzerland) of pH = 4.01 ± 0.02, pH = 7.00 ± 0.02 and pH = 9.21 ± 0.02 at T = 23.0 ± 2.0 °C.

#### 2.5.3. Gel Permeation Chromatography (GPC) Analysis

The number average molar masses (M_n_) of the PLA and PHA welded samples was estimated by GPC experiments conducted in chloroform at 35 °C and a flow rate of 1 mLmin^−1^ using a Spectra-Physics 8800 solvent delivery system with two Mixed C Styragel columns in series and a Shodex SE 61 refractive index detector. A 10 μL volume of the sample solutions in chloroform (0.5% *w*/*v*) was injected into the system. Polystyrene standards with low dispersity were used to generate a calibration curve [27].

#### 2.5.4. ^1^H Nuclear Magnetic Resonance (NMR) Spectroscopy

^1^H-NMR spectra were recorded using a Bruker-Advance spectrometer operating at 600 MHz with Bruker TOPSPIN 2.0 software using CDCl_3_ as the solvent and tetramethylsilane (TMS) as the internal standard. Spectra were obtained with 64 scans, a 11 μs pulse width, and a 2.66 s acquisition time [28].

#### 2.5.5. Electrospray Ionization Mass Spectrometry (ESI-MS) Analysis

ESI-MS experiments of the degradation medium were performed using a Finnigan LCQ ion trap mass spectrometer (Finnigan, San Jose, CA, USA). The samples after the degradation process were dissolved in methanol/water system (1:1; *v*/*v*) and then introduced to the ESI source by continuous infusion using the instrument syringe pump with the rate at 6 mL/min. The ESI source was operated at 4.50 kV and the capillary heater was set to 200 °C. The analysis was performed in positive-ion mode. Nitrogen was used as the sheath gas; helium was used as the auxiliary gas.

#### 2.5.6. Scanning Electron Microscopy (SEM)

Microscopic changes of the surface of the PHA 3 sample were analyzed by the Scanning Electron Microscopy (SEM) method. The samples were analyzed without coating under a low vacuum (80 Pa) by using a large field detector. SEM studies were performed using the Quanta 250 FEG (FEI Company, Fremont, CA, USA) high-resolution environmental scanning electron microscope operated at 10 kV acceleration voltage.

## 3. Results and Discussion

### 3.1. Structural Studies of The PLA and PHA Samples and Their Welds by ^1^H-NMR and GPC Analyses

**Figure 1:**
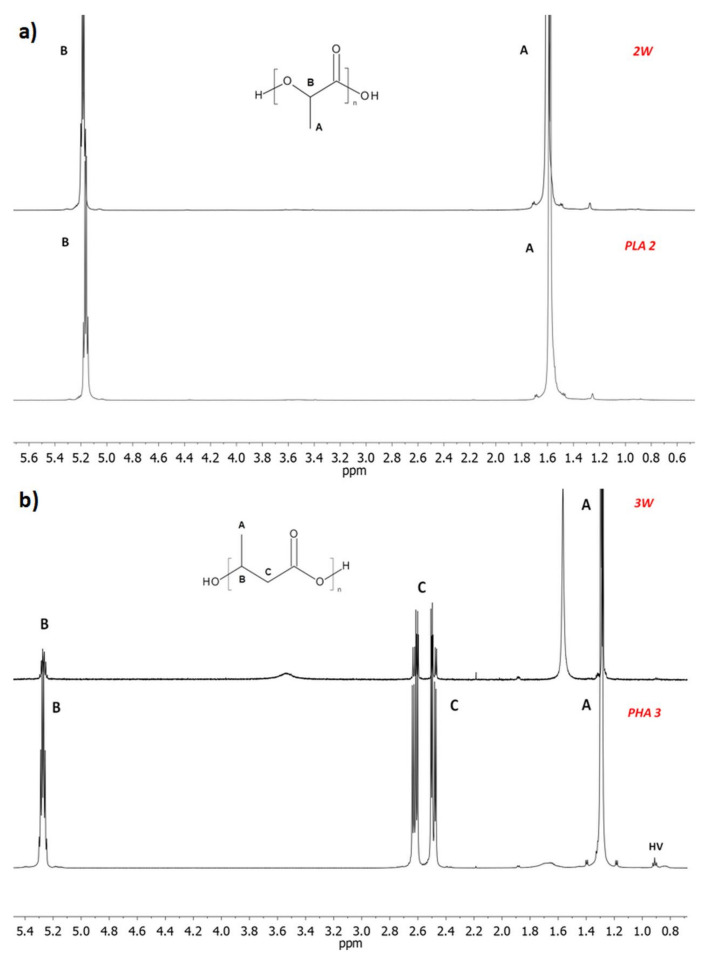
^1^H-NMR spectra of samples (**a**) PLA 2 and (**b**) PHA 3 and of their welds (2W and 3W).

**Figure 2:**
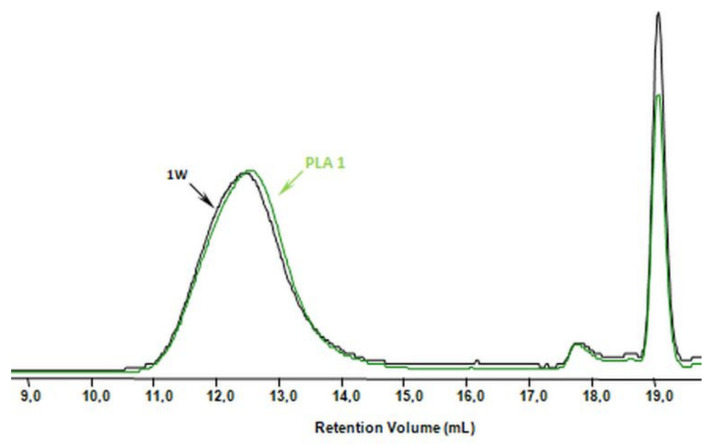
GPC traces of sample PLA 1 before and after welding (1W).

The influence of the welding process on the chemical structure of the samples was investigated by ^1^H-NMR and GPC analyses. Figure 1a,b compares the ^1^H-NMR spectra of PLA and PHB samples before welding (samples PLA 2 and PHA 3) and their corresponding welds (samples 2W and 3W, respectively). In the ^1^H-NMR spectra of the PLA 2 sample and its weld (sample 2W), Figure 1a, the presence of the signals corresponding to the protons of methyl groups at 1.56 ppm (A) and methine groups at 5.16 ppm (B), characteristic of lactide repeating units, was observed [22]. Similar results were obtained for PLA 1 and its weld sample 1W (data not shown). The ^1^H-NMR spectra of sample PHA 3 and its weld 3W (Figure 1b) revealed the presence of the characteristic signals corresponding to the protons of methyl groups at 1.28 ppm (A), methylene groups at 2.54 (C) and methine groups at 5.26 (B) of 3-hydroxybutyrate repeating units. Moreover, the low intensity signal at 0.9 ppm, which corresponds to the protons of methyl groups of 3-hydroxyvalerate repeating units, was also observed [29]. The NMR study indicated that the chemical structure of the PLA and PHA samples determined before and after the welding process remained unchanged. It can, therefore, be assumed that the welding methods used did not affect the chemical structure of the tested PHA and PLA samples. Moreover, no significant differences on average molar masses of the PLA and PHA samples before and after the welding process were observed. Figure 2 shows an example the GPC traces for the sample PLA 1 and its weld sample 1W.

### 3.2. Thermal Analysis of The Samples and Their Welds before The Hydrolytic Degradation Process

**Figure 3:**
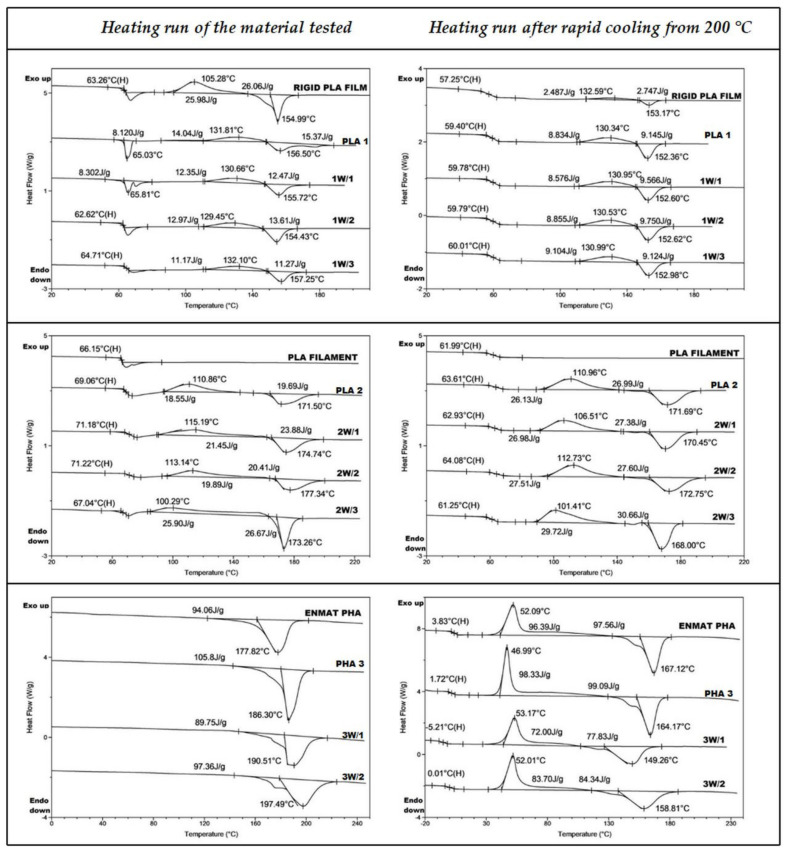
DSC traces of the samples tested.

To evaluate the changes in the thermal properties, DSC analysis of the tested samples before and after welding was conducted. The results are summarized in Table 2 and Figure 3. All studies for the samples of welds 1W, 2W and 3W were performed in triplicate. The thermal properties of the respective weld were expressed as the mean and standard deviation in Table 2. These values are compared with the thermal properties of the samples before welding (samples PLA 1, PLA 2 and PHA 3), as well as with the thermal properties of the starting material (PLA rigid film prepared from commercial polylactide type 2002D used for the formation of the PLA 1 sample, PLA filament used for the formation of the PLA 2 sample, and ENMAT Y1000 biopolyester used for preparation of PHA 3 sample).

The DSC analysis of the PLA rigid film, PLA 1 and 1W samples indicated that the weld, the sample before welding and the starting material (PLA rigid film) were amorphous (Table 2, Figure 3). The heating DSC trace for the PLA rigid film and 1W samples shows a small endothermic phenomenon, the structural relaxation, overlapped with the glass transition near 63 °C. For sample PLA 1, *T_g_* wasn’t detected (see Figure 3). Further heating of all samples showed cold crystallization and melting endotherms (see PLA rigid film, PLA 1 and 1W samples, Table 2, Figure 3) [30]. The determined enthalpy values of consecutive processes of cold crystallization and melting were similar, suggesting that the samples were amorphous, as evidenced by DSC. This means that PLA 1 and its weld have similar thermal properties, so the ultrasonic welding method did not affect the thermal properties. It is known that in ultrasonic welding heating is confined to the welding interface and heat degradation of the polymers is minimized [31].

The DSC trace of the starting PLA filament shows only a glass transition temperature that confirms the amorphous microstructure of this sample [24]. The PLA 2 and its weld 2W also show glass transition temperature (Figure 3). However, further heating of the PLA 2 and 2W samples above the glass transition temperature exhibit the so-called “cold crystallization” phenomenon which was not observed for the starting PLA filament (see Table 2, Figure 3). It seems that the observed phenomena in the case of samples PLA 2 and 2W, can be explained by a crystallization and/or nucleation processes that can be initiated by PLA oligomers which could form during the 3D-printing of PLA 2 sample [24,25,26,27,28].

In the case of ENMAT PHA, PHA 3 and 3W samples the DSC heating runs show that all the samples are crystalline (Table 2). Significantly different melting points for ENMAT PHA, PHA 3 and 3W samples were observed (see Table 2, Figure 3). Welding at a temperature of about 200 °C can influence the recrystallization process of existing crystal structure and lead to a more ordered structure that is revealed by the *T_m_* value increase of the samples studied (see Table 2, Figure 3). Moreover, the decrease in the *T_g_* of PHA 3 and 3W samples observed indicates on their partial thermal degradation lead to the gradual decrease in their molar masses [32]. Thus, the application of the hot press processing method as well as the heated tool welding method influences the thermal properties of PHA-based samples.

### 3.3. Hydrolytic Degradation Studies

To investigate the impact of the welding methods used on the stability of the obtained welded joints of the PLA and PHA biopolymer samples, hydrolytic degradation tests were carried out. The progress of the degradation process was monitored by pH changes in the degradation medium, as well as the macroscopic changes of the surface of welded samples [27,33].

**Figure 4:**
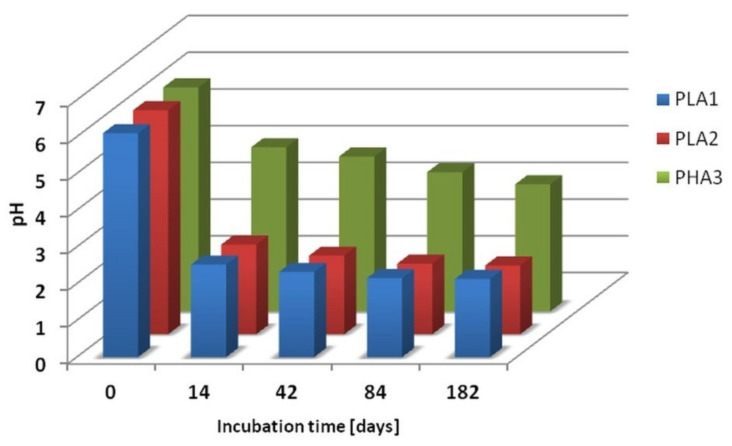
pH changes of the degradation medium before and after 14, 42, 84 and 182 days of incubation of PLA 1 PLA 2 and PHA 3 welded samples.

**Figure 5:**
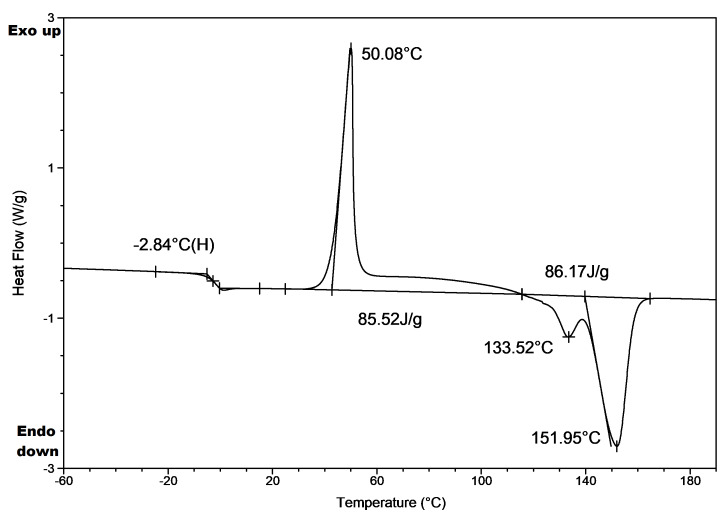
DSC heating trace obtained after rapid cooling from 200 °C, for PHA 3 sample residues remaining after 9 months of hydrolytic degradation.

Figure 4 shows the changes in the pH of the degradation media versus incubation time of the PLA 1, PLA 2 and PHA 3 samples. The observed decrease of the pH of the degradation medium was caused by the hydrolytic degradation of the PLA and PHA polyester samples and the appearance of the degradation products in the water medium. When the molar mass of PLA and PHA welded samples drops below a certain value (M_n_ lower than 700 g/mol), the oligomers become water-soluble and diffuse into the surrounding medium. For all samples studied, a decrease of the pH values in the degradation media was observed, on account of the release of lactic or 3-hydroxybutyric acids and their low molar masses PLA and PHA oligomers terminated by hydroxyl and carboxyl end groups released from the tested samples into the water medium. This is accompanied by the disintegration of the tested solid samples (see further paragraphs). The smallest changes in pH were observed for sample PHA 3, due to the lower degradation rate of PHA than the PLA biopolymer. [26] However, DSC analysis of PHA 3 sample residues remaining after 9 months of hydrolytic degradation shows a decrease in *T_g_* from 1.7 to −2.8 °C (see Table 2 and Figure 5), which indicates the presence of oligomers inside the polymer matrix of sample PHA 3 and confirms the hydrolysis of PHA polyester chains.

### 3.4. Macroscopic Changes in The Samples Caused by Hydrolytic Degradation

**Figure 6:**
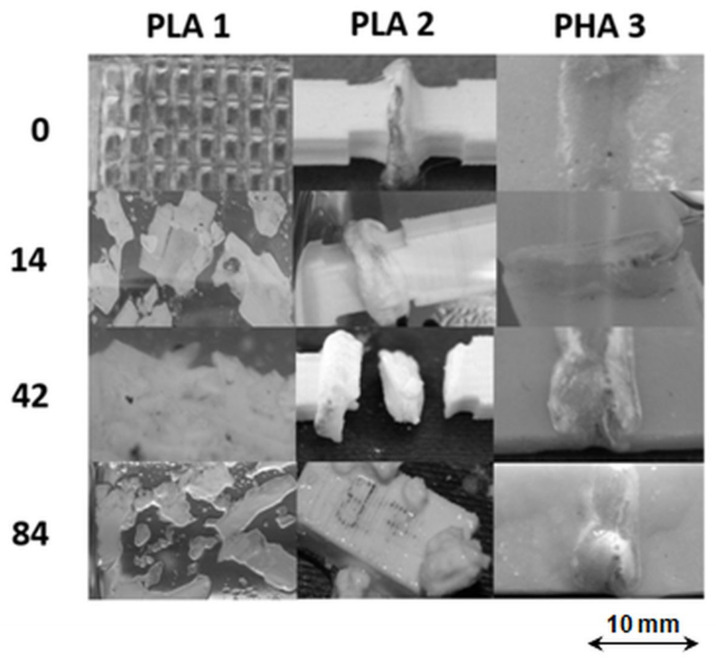
Photographs of welded samples before (0) and after 14, 42 and 84 days of incubation in water at 70 °C.

Figure 6 presents the photographs of samples after hydrolytic degradation in water at 70 °C after the specified time period in days.

**Figure 7:**
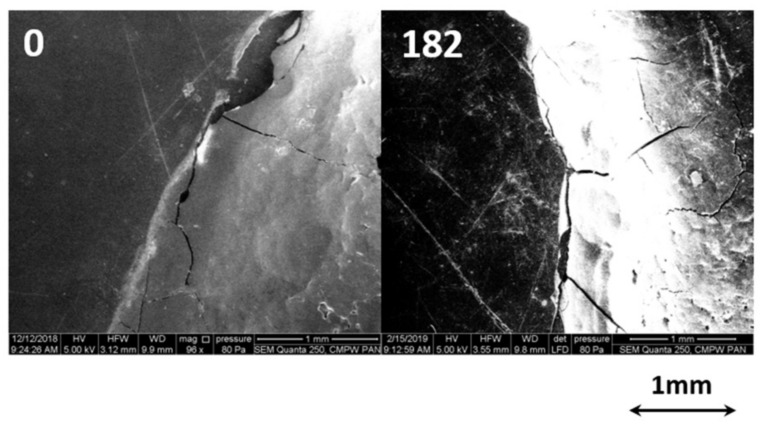
SEM images (96×) of the surface of PHA 3 before and after 182 days of incubation in water at 70 °C.

The images show that the welds were well-formed before hydrolytic degradation. After 14 days of incubation, stratification of sample PLA 1 was noticed. The observed disintegration process seems to go faster in the weld (1W), compared to other parts of PLA. After 84 days of incubation, PLA 1 completely lost its integrity and the degradation medium became turbid. In contrast, the surface of PLA 2 and PHA 3 and their welds remain unchanged for 14 days of hydrolytic degradation. After 42 days of incubation, the loss of weld cohesion of the weld was noticed in PLA 2; in the case of sample PHA 3, the weld bulges above the surface. However, it is worth noting that the parts of PLA 2 and PHA 3 in the vicinity of their welds did not disintegrate at that time. During further incubation, PLA 2 became swollen and prone to cracking, which resulted in the progression of disintegration of the sample and its disappearance after 182 days of degradation, similar to what was observed for PLA 1. In the case of PHA 3, the start of swelling of the weld was observed after 42 days of incubation while damages in the vicinity of the weld were only noticed after 182 days of incubation. The microscopic changes in PHA 3 were investigated using SEM analysis. Figure 7 shows the SEM images of PHA 3 before and after 182 days of incubation in water at 70 °C.

As can be seen from the images of PHA 3 before degradation, weld cracks can be observed. This may be due to the internal tension or thermal treatment during welding. The visibly lighter color of the weld after 182 days of incubation indicates that the weld protrudes over the surface, which is in agreement with the macroscopic observation. The cracking process of the weld progresses during the incubation time, which may suggest faster degradation of the weld than the rest of PHA 3.

### 3.5. Determination of The Chemical Structure of Hydrolytic Degradation Products of Biopolyester Welds by ESI-MS Analysis

The progress of the hydrolytic degradation process was also monitored by the analysis of the degradation media. It is well known that the hydrolysis of PLA and PHA occurs via random ester bond cleavage along the polyester chains which leads to the formation of shorter polyester chains. When the molar mass of the degraded PLA and PHA welded samples drops below a 700 g/mol, the oligomers become water-soluble and diffuse into the surrounding medium. To determine the chemical structure of water-soluble products formed during the hydrolytic degradation of PLA and PHA welded samples, mass spectrometry (ESI-MS) was used. The application of this technique in the structural studies of degradation products allowed us to detect even small amounts of the degradation products. Recently, ESI-MS techniques have been successfully used for the structural characterization at the molecular level of the degradation products of aliphatic polyesters [34,35].

**Figure 8:**
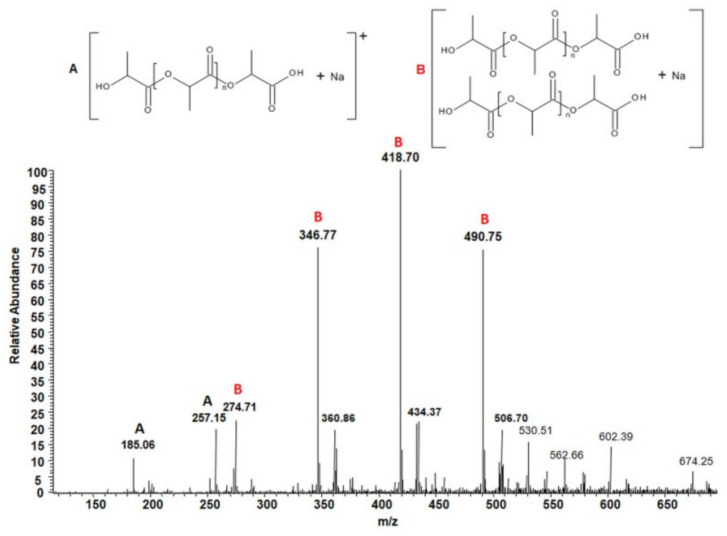
ESI-mass spectrum (positive-ion mode) of the degradation products released from the welded PLA 2 to the water after 9 months of hydrolysis.

The purpose of this research was to check whether the structure of degradation products released from welded PLA and PHA samples is the same as that previously observed in the case of unwelded PLA and PHA biopolyesters. Figure 8 shows the positive ESI-mass spectrum of the low molar mass compounds released from the welded PLA 2 to the water after 9 months of hydrolysis.

Singly charged signals with a peak-to-peak mass increment of 72 Da, which is equal to the molar mass of the lactic acid repeating unit, were observed in this spectrum. The signals observed correspond to the sodium adducts of water-soluble lactic acid oligomers terminated by carboxyl and hydroxyl end groups (see the structures in Figure 8). Thus, lactic acid oligomers terminated by hydroxyl and carboxyl end groups were identified as water-soluble degradation products of welded PLA 2 samples. This observation was in agreement with the results of the hydrolytic degradation of PLA after multiple extrusion as previously reported [27,30,31,32,33,34,35,36].

**Figure 9:**
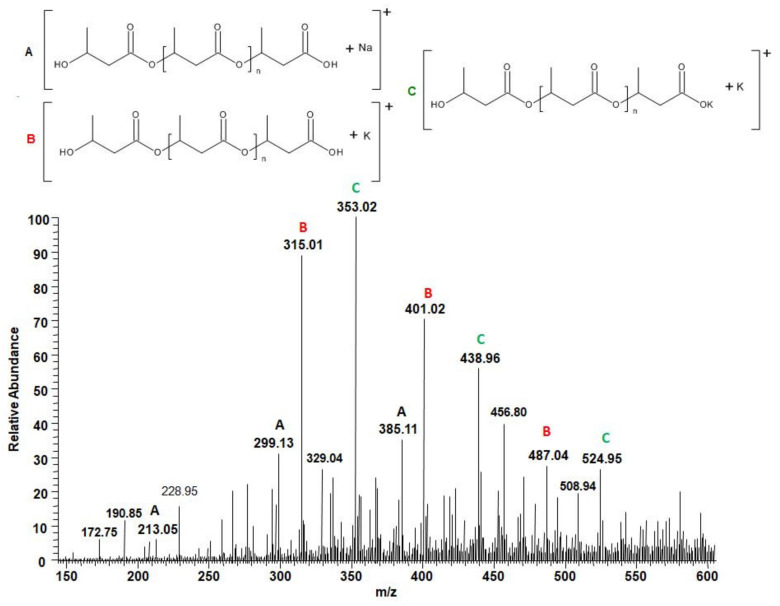
ESI-mass spectrum (positive-ion mode) of the degradation products released from welded PHA 3 to the water after 9 months of hydrolysis.

Figure 9 shows the positive ESI-MS spectrum of the low molar mass compounds released from the welded PHA 3 sample to the water after 9 months of hydrolysis.

Singly charged signals with a peak-to-peak mass increment of 86 Da were observed in the spectrum obtained for the water-medium after hydrolytic degradation of the welded sample of PHA 3. The signals observed correspond to the sodium and potassium adducts of water-soluble 3-hydroxybutyrate oligomers, separated by 86 Da, which is equal to the molar mass of the 3-hydroxybutyrate repeat unit. Thus, 3-hydroxybutyrate oligomers with hydroxyl and carboxyl end groups were identified as water-soluble degradation products of welded PHA 3 samples. This observation was in agreement with the results of the hydrolytic degradation of PHA observed earlier [37].

## 4. Conclusions

Herein, we report the impact of the welding process on the structure and properties of PLA and PHA biopolymer welds and their hydrolytic degradation for the first time. The small changes in the thermal properties of PLA and PHA samples in the region of the weld were only observed when the heated tool method was used. The hydrolytic degradation tests performed indicated that all the samples studied degrade during incubation in water but with a different rate. The rate of degradation of the samples PLA (1 and 2) was higher than for the sample PHA (3). In the case of the PLA and PHA samples, the degradation process started in the region of the weld. The structural studies of the products released to the water were carried out with the aid of ESI-MS spectrometry. The lactic acid or 3-hydroxybutyrate oligomers terminated by hydroxyl and carboxyl end groups were identified as water-soluble degradation products for samples PLA (1 and 2) and PHA (3), respectively. No changes were observed in the structure of products released to media during hydrolytic degradation of welded materials when compared with the degradation products of unwelded PLA and PHA. The current study demonstrated the need for precise evaluation of the structure, properties, and behavior of advanced biodegradable polymer packing materials in order to avoid potential failures of the commercial products to be manufactured from them.

## Figures and Tables

**Table 1:** 3D-printing parameters.

3D-Printer Settings	
Extruder temperature, °C	200
Platform temperature, °C	50
Layer thickness, mm	0.14
First layer thickness, mm	0.21
Printing speed, mm/s	80
Moving speed, mm/s	110
Filling, %	100
Filling form	line

**Table 2:** Selected thermal properties of tested samples. Heating rate 20 °C/min.

	PLA Rigid Film	PLA 1	1W	PLA Filament	PLA 2	2W	ENMAT PHA	PHA 3	3W
**Heating Run of The Materials Tested**									
*T_g_* [°C]	63.3	n.d.	63.7 ± 1.5	66.2	69.1	69.8 ± 2.4			
*T_cc_* [°C]	105.3	131.8	130.7 ± 1.3	-	110.9	109.5 ± 8.1	-	-	-
*ΔH_cc_* [J/g]	26.0	14.0	12.2 ± 0.9	-	18.6	22.4 ± 3.1	-	-	-
*T_m_* [°C]	155.0	156.5	155.8 ± 1.4	-	171.5	175,1 ± 2.1	177.8	186.3	194.0 ± 4.9
*ΔH_m_* [J/g]	26.1	15.4	12.5 ± 1.2	-	19.7	23.7 ± 3.1	94.1	105.0	93.6 ± 5.4
*X_c_* [%]	-	-	-	-	-	-	57.4	64.0	57.1
**Heating Run after Rapid Cooling from 200 °C**									
*T_g_* [°C]	57.3	59.4	59.9 ± 0.1	62.0	63.6	62.8 ± 1.4	3.8	1.7	−2.61 ± 3.7
*T_cc_* [°C]	132.6	130.3	130.8 ± 0.3	-	110.9	106.9 ± 5.7	52.1	47.0	52.6 ± 0.8
*ΔH_cc_* [J/g]	2.5	8.8	8.8 ± 0.3	-	26.1	28.1 ± 1.5	96.4	98.3	77.8 ± 8.3
*T_m_* [°C]	153.2	152.4	152.7 ± 0.2	-	171.7	170.4 ± 2.4	167.1	164.2	154.1 ± 6.8
*ΔH_m_* [J/g]	2.7	9.1	9.5 ± 0.3	-	27.0	28.5 ± 1.8	97.6	99.1	81.1 ± 4.6
*X_c_* [%]	-	-	-	-	-	-	59.5	60.4	49.4

*T_g_*—glass transition temperature, *T_m_*—melting temperature, *ΔH_m_*—melting enthalpy, *T_cc_*—maximum of the exothermic peak of the cold crystallization temperature, *ΔH_cc_*—cold crystallization enthalpy, *X_c_* —degree of crystallinity 1W, 2W and 3W—the welds from the PLA 1, PLA 2 and PHA 3 samples respectively; PLA 1, PLA 2 and PHA 3—samples before welding. PLA rigid film—starting material prepared from commercial polylactide 2002D used for the formation of sample PLA 1, PLA filament-starting material used for the formation of PLA 2 sample, and ENMAT PHA-starting material used for preparation of PHA 3 sample.
